# AbobotulinumtoxinA using 2-mL dilution (500 U/2-mL) maintains durable improvement across multiple treatment cycles

**DOI:** 10.1186/s40734-020-00090-x

**Published:** 2020-08-31

**Authors:** Khashayar Dashtipour, Stefan Wietek, Bruce Rubin, Pascal Maisonobe, Laxman Bahroo, Richard Trosch

**Affiliations:** 1 Loma Linda University School of Medicine, Loma Linda, CA 92350 USA; 2 Ipsen, Cambridge, MA 02142 USA; 3 Formerly of Ipsen, Cambridge, MA 02142 USA; 4 Ipsen Pharma, 92100 Boulogne-Billancourt, France; 5 Georgetown University Hospital, Washington, DC 20007 USA; 6 Parkinson’s and Movement Disorders Center, Farmington Hills, MI 48334 USA

**Keywords:** AbobotulinumtoxinA, Safety, Efficacy, Cervical dystonia, Open-label extension study, Repeat treatments

## Abstract

**Background:**

Cervical dystonia (CD), the most common focal dystonia, is a chronic neurological movement disorder characterized by sustained involuntary contractions of the neck muscles, leading to abnormal postures. AbobotulinumtoxinA (aboBoNT-A) was approved in the US initially as a 500 U per 1-mL dilution and subsequently, as a 500 U/2-mL dilution (or 250 U/mL), thereby providing clinicians with more flexible dosing options to better meet individual patient needs. The objective of this open-label extension study was to evaluate the longer term safety and efficacy of repeat treatments with aboBoNT-A using 2-mL dilutions in adults with cervical dystonia.

**Methods:**

Patients (*N* = 112) from a 12-week, double-blind lead-in study (NCT01753310) received up to three additional treatments of aboBoNT-A, with re-treatment every 12–16 weeks based on clinical judgment. Safety was assessed through treatment-emergent adverse events (TEAEs). The Toronto Western Spasmodic Torticollis Rating Scale (TWSTRS) total and subscale scores were measured at day 1 of each treatment cycle (C), 4 weeks after each treatment, and 12 weeks after the third treatment. Descriptive statistics were used for all analyses.

**Results:**

In cycles 1, 2, 3, and 4, respectively, 35.7, 25.9, 30.2, and 22.8% of patients reported TEAEs. Dysphagia, muscular weakness, and neck pain were each reported by 10.7% of patients, over the full study duration. Mean TWSTRS total score decreased from 37.7 (SD 13.6 [C1, day 1]) to 30.1 (SD 12.8 [C3, week 12]). In each cycle, TWSTRS total and subscale scores decreased from day 1 to week 4 and increased between weeks 4 and 12, though the week 12 scores remained lower than day 1 scores.

**Conclusion:**

Extended treatment of cervical dystonia with aboBoNT-A (up to 3 additional treatment cycles) using a 2-mL dilution is effective, with a positive risk-benefit profile.

**Trial registration:**

ClinicalTrials.gov Identifier: NCT01753336. Registered 17 Dec 2012.

## Introduction

Cervical dystonia (CD), the most common focal dystonia, is a chronic neurological movement disorder characterized by sustained involuntary contractions of the neck muscles, leading to a disabling posture [1]. The epidemiology of CD is not well characterized due to highly variable study designs and study populations [2]. However, a review of insurance records for a racially diverse population in California found the minimum estimated overall incidence of CD to be 8 per million person-years, with the preponderance of cases occurring among whites versus other races (12.3 vs. 1.5 per million person-years) [3]. Moreover, women were twice as likely as men to be diagnosed with CD. Although the number of affected patients is relatively small, with few treatment options, the disabling impact of this disorder can be great for patients and their families.

AbobotulinumtoxinA, one of four currently approved botulinum toxin preparations for treatment of CD, received the indication using a 500 U/1-mL dilution in the United States of America in 2009. Approval was based on two pivotal studies [4, 5] and their respective open-label extension studies [6]. To provide clinicians with more flexible dosing to better meet individual patient needs, the safety and efficacy of a 500 U/2-mL dilution (or 250 U/mL) was investigated. The subsequent approval of the 2-mL dilution was based on results of a 12-week, phase 3b, multicenter, randomized, double-blind, placebo-controlled trial in adults with CD (NCT01753310) [7]. This study demonstrated that abobotulinumtoxinA significantly improved symptoms in both toxin-naïve and previously treated patients, with a safety profile consistent with the 1-mL dilution. In this open-label extension (OLE) of the 12-week, double-blind study (NCT01753336), we assess the longer term safety and efficacy of abobotulinumtoxinA using a 2-mL dilution in adults with CD for up to 3 additional treatment cycles.

## Methods

### Study design

**Fig. 1:**
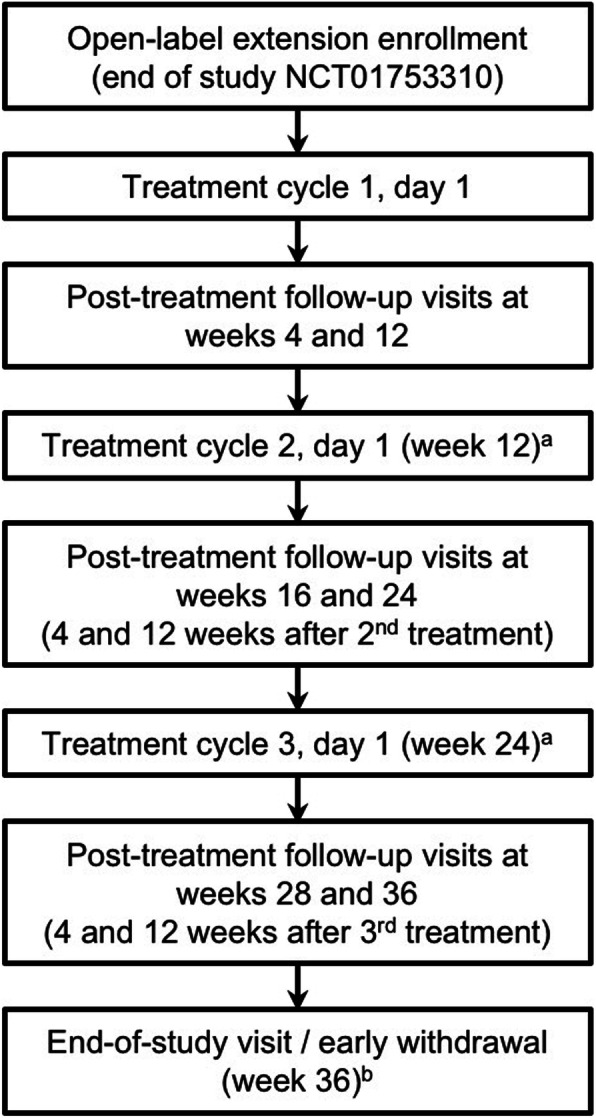
Study Flow Chart. ^a^This visit should occur on the same day as the last visit of the previous treatment cycle. Re-treatment was expected to occur approximately 12–16 weeks after the last treatment; however, the exact timing of treatment was determined by the investigator based on clinical need. ^b^Patients who withdrew from the study early (ie, before week 12 of cycle 3) underwent all procedures required for the week 36 visit

This was a phase 3b, prospective, multicenter, OLE study to a double-blind, placebo-controlled lead-in study (NCT01753310) [7] that evaluated the efficacy and safety of a single cycle of abobotulinumtoxinA using a 2-mL dilution in adults with CD. The current study incorporated a maximum of three additional treatment cycles, occurring at intervals of no less than 12 weeks, a follow-up visit 4 weeks after each treatment, and an end-of-study visit 12 weeks after the third treatment visit (Fig. 1). Patients in the lead-in placebo group had exposure of up to three cycles, whereas those in the lead-in abobotulinumtoxinA group had overall exposure of up to four treatment cycles.

The OLE study was conducted under the provisions of the Declaration of Helsinki and in accordance with the International Conference on Harmonisation (ICH) Consolidated Guideline on Good Clinical Practice. In addition, this study adhered to relevant company policies and all U.S. Food and Drug Administration (FDA) regulatory requirements. Study protocol and amendments were approved by an independent Institutional Review Board and all patients or their legal representative signed an informed consent form.

Adult patients who completed the week 12 visit of the lead-in study, or patients who withdrew after week 4 and whose TWSTRS [8] total score between weeks 4 and 8 was reduced by ≤15% from baseline, were invited to participate in this OLE study.

### Treatment

The OLE incorporated up to three additional treatment cycles, with re-treatment occurring every 12 to 16 weeks based on clinical judgment. Day 1, OLE cycle 1 was the same as the last lead-in study visit. For OLE cycle 1, patients from the placebo group at lead-in baseline received abobotulinumtoxinA 500 U/2 mL in ≥2 affected neck muscles and patients from the treatment group at lead-in baseline received the same starting dose of abobotulinumtoxinA dose (250–500 U/2 mL) received in the lead-in study injected into previously treated muscles. For cycles 2 and 3, the need for dose adjustments was determined by the investigator, with allowed increments and decrements limited to ≤250 U/cycle and maximal total dose limited to 1000 U/cycle.

### Safety and tolerability

Safety and tolerability were assessed by recording TEAEs, as well as through physical examinations, vital signs, and clinical laboratory assessments. These assessments were performed on day 1 of each treatment cycle, which occurred 12 to 16 weeks apart, and at the last study visit or early withdrawal visit.

### Efficacy

Efficacy assessments were based on TWSTRS total and subscale (severity, disability, and pain) scores. Scores could range from 0 to 35, 0 to 30, and 0 to 20, for the severity, disability, and pain subscales, respectively. TWSTRS total scores range from 0 (best) to 85 (worst) and comprise the sum of the three individual scores’ subscales. TWSTRS assessments were conducted by the investigator prior to the administration of study treatment at baseline (day 1 of treatment cycle 1) and at posttreatment visits.

### Statistical analyses

All analyses are descriptive in nature and were conducted using the safety population, which included all patients who received ≥1 dose of study treatment and had ≥1 safety record post treatment. Adverse events were assigned to a specific cycle based on the first exposure to abobotulinumtoxinA; therefore, patients who received abobotulinumtoxinA in the lead-in study had four cycles, whereas patients who received placebo in the lead-in study had three cycles. Statistical analyses of efficacy and safety data were performed using Statistical Analysis System (SAS)^®^ (version 9.4).

## Results

### Patients and dosing

**Fig. 2:**
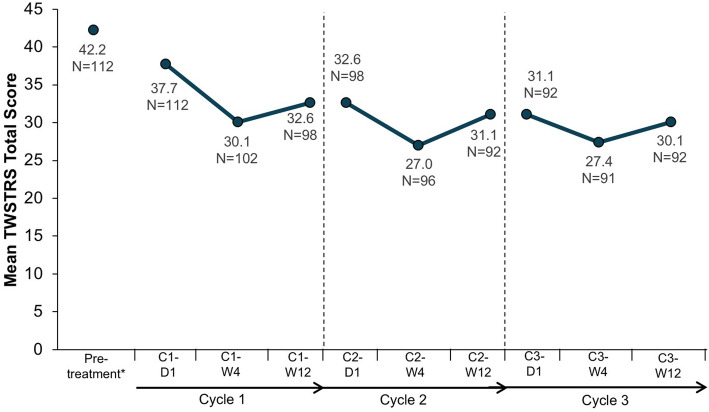
Change from baseline in TWSTRS total score. C, cycle; D, day; W, week. *Before first abobotulinumtoxinA administration

A total of 112 patients were enrolled and treated, and 92 (82.1%) completed the week 36 visit (cycle 3, week 12) (Fig. 2). No patients were excluded from the safety analysis set; 20 patients (17.9%) withdrew from the study due to personal reasons (*n* = 12), lost-to-follow-up (*n* = 3), investigator’s decision (*n* = 2), sponsor’s decision (*n* = 2), or other (*n* = 1). No patients withdrew due to an adverse event. Cycle durations lasted approximately 13 weeks (Cycle 1, median 12.0 [range 3–29]; Cycle 2, median 12.0 [range 8–15]; Cycle 3, median 13.0 [range 8–58).

Of the 112 patients enrolled, 77 (69%) had prior neurotoxin exposure (non-naïve), and 35 (31%) had no neurotoxin exposure prior to entering the lead-in study (toxin-naïve). Patients’ median age was 56 (range 29–82) years, 94.6% were white, and 62.5% were female (Table 1). The mean body mass index was 27.0 (± 4.9) kg/m^2^.

**Table 1:** Demographics and baseline characteristics

	Patients (*N* = 112)
Toxin-naïve	35 (31.3%)
Non-naive	77 (68.8%)
Age, years	
Mean	57.2 (11.0)
Median (range)	56 (29, 82)
Sex, *n* (%)	
Male	42 (37.5%)
Female	70 (62.5%)
BMI, kg/m^2^	
Mean (SD)	27.0 (4.9)
Median	26
Range	18–46
Race, *n* (%)	
White	106 (94.6%)
Black	2 (1.8%)
Asian	3 (2.7%)
Multiple	1 (0.9%)
Ethnicity, *n* (%)	
Hispanic or Latino	16 (14.3%)
Not Hispanic or Latino	96 (85.7%)

*BMI* Body mass index, *SD* Standard deviation

The total dose and volume of abobotulinumtoxinA administered by treatment cycle, with overall results, are summarized in Table 2. The median dose administered by treatment cycle was 500 U and ranged from 200 U to 500 U in cycle 1; 150 U to 806 U in cycle 2; and 163 U to 1000 U in cycle 3 in all patients. The mean total dose increased over treatment cycles, with toxin-naïve patients receiving higher doses on average than did non-naïve patients. Median dose for toxin-naïve patients was 1500 U, ranging from 500 U to 2250 U, and 1444 U in non-naïve patients, ranging from 263 U to 2031 U. The mean dose in the OLE third cycle was 596.5 U (SD 192.4) for toxin-naïve patients and 494.5 U (SD 143.4) among patients with prior exposure. A median of five muscles were injected at five sites for all patients and non-naïve patients during all treatment cycles and for naïve patients at treatment cycle 1. At treatment cycles 2 and 3, naïve patients had injections in a median of six muscles at six sites. The most frequently injected muscle was the splenius capitis for 88.4, 89.7, and 90.3% of patients during treatment cycles 1, 2, and 3, respectively (Table 3). More than 50% of patients had injections in their sternocleidomastoid, trapezius, and levator scapulae muscles in each treatment cycle.

**Table 2:** AbobotulinumtoxinA administration by treatment cycle

	Treatment Cycle 1	Treatment Cycle 2	Treatment Cycle 3	All Treatment Cycles
All patients, *n*	112	97	93	112
Total dose, administered in Units, mean (SD)	457.7 (75.3)	507.1 (130.3)	527.4 (166.8)	1334.8 (476.9)
Total dose <500 U, *n* (%)	38 (33.9%)	31 (32.0%)	30 (32.3%)	–
Total dose = 500 U, *n* (%)	74 (66.1%)	41 (42.3%)	38 (40.9%)	–
Total dose >500 U, *n* (%)	0	25 (25.8%)	25 (26.9%)	–
Toxin-naïve patients, *n*	35	31	30	35
Total dose, administered in Units, mean (SD)	492.9 (34.2)	549.5 (114.6)	596.5 (192.4)	1490.9 (468.6)
Total dose <500 U, *n* (%)	2 (5.7%)	5 (16.1%)	5 (16.7%)	–
Total dose = 500 U, *n* (%)	33 (94.3%)	15 (48.4%)	15 (50.0%)	–
Total dose >500 U, *n* (%)	0	11 (35.5%)	10 (33.3%)	–
Non-naïve patients, *n*	77	66	63	77
Total dose, administered in Units, mean (SD)	441.7 (83.2)	487.1 (133.2)	494.5 (143.4)	1263.8 (466.5)
Total dose <500 U, *n* (%)	36 (46.8%)	26 (39.4%)	25 (39.7%)	–
Total dose = 500 U, *n* (%)	41 (53.2%)	26 (39.4%)	23 (36.5%)	–
Total dose >500 U, *n* (%)	0	14 (21.2%)	15 (23.8%)	–

Drug exposure for each cycle was calculated as the sum of all doses within a cycle, across all injection sites. Total cumulative drug exposure was the sum of doses across all treatment cycles. *SD* Standard deviation, *U* Unit

**Table 3:** AbobotulinumtoxinA administration by muscle group and cycle

	Treatment Cycle 1 ***N*** = 112	Treatment Cycle 2 ***N*** = 97	Treatment Cycle 3 ***N*** = 93
**Muscles injected, n (%)**			
Splenius capitis	99 (88.4)	87 (89.7)	84 (90.3)
Sternocleidomastoid	75 (67.0)	67 (69.1)	63 (67.7)
Trapezius	73 (65.2)	66 (68.0)	62 (66.7)
Levator scapulae	67 (59.8)	60 (61.9)	57 (61.3)
Scalenus (medius and anterior)	29 (25.9)	31 (32.0)	28 (30.1)
Semispinalis capitis	38 (33.9)	39 (40.2)	25 (37.6)
Longissimus	23 (20.5)	24 (24.7)	21 (22.6)
Other	29 (25.9)	26 (26.8)	24 (25.8)

### Safety and tolerability

A total of 220 TEAEs were reported among 70 patients (62.5%) (Table 4). The percentages of patients reporting TEAEs in cycles 1, 2, 3, and 4 were 35.7, 25.9, 30.2, and 22.8%, respectively. Dysphagia, muscular weakness, and neck pain were the most frequent TEAEs (10.7% each). Of the 12 patients who reported dysphagia; 6 patients received injections into the sternocleidomastoid muscle with doses ranging from 50 U to 250 U, 3 patients received no injections into the sternocleidomastoid muscle, and 1 patient had 2 incidences of dysphagia, once after receiving a 75 U injection and once without an injection into the sternocleidomastoid muscle. Details regarding muscles injected were unavailable for 2 patients. Most TEAEs (*n* = 156/220; 70.9%) were not considered by the investigators to be treatment related.

**Table 4:** Summary of safety and tolerability across all treatment cycles

Patients, *n* (%)	Patients (*N* = 112)
Any TEAE Intensity	70 (62.5%)
Severe	10 (8.9%)
Moderate	38 (33.9%)
Mild	53 (47.3%)
Serious adverse events (SAEs)	7 (6.3%)
Treatment-related SAE	33 (29.5%)
Not related	56 (50.0%)
Missing	1 (0.9%)
Leading to study withdrawal	0
Leading to death	0

*TEAE* Treatment-emergent adverse event

Seven patients reported eight serious TEAEs. There were two episodes of osteoarthritis (severe) and one each of: appendicitis, cholecystitis, dysphagia (severe), ischemic colitis (severe), thoracic vertebral fracture, and transient ischemic attack (severe). One serious TEAE (dysphagia), which occurred during treatment cycle 1, three days after receiving the first dose of 500 U/2-mL dilution of abobotulinumtoxinA, was judged to be severe and treatment related. The injections had been made into the right splenius capitis muscle, left splenius capitis, sternocleidomastoid, levator scapulae and trapezius muscles. This event occurred in a 56-year-old, white female who had torticollis, laterocollis, and a history of dysphagia at enrollment to the lead-in study. The patient presented with great difficulty with swallowing and was unable to eat or drink. During an esophagogastroduodenoscopy 5 days later, a small Schatzki’s ring in the distal esophagus was found. Although the small Schatzki ring could have been partly responsible for the presented symptoms, it was reported that the event may have been an underlying condition that was brought to light due to study drug administration. This event lasted from August 21st to October 1st, and the patient received the next dose of abobotulinumtoxinA on November 19th. Other reported serious TEAEs were considered not to be treatment related. None of the TEAEs led to study withdrawal or death. All patients recovered; one patient had sequelae following the severe, but not-related, event of a transient ischemic attack (TIA) that occurred during treatment cycle 1.

### Efficacy

**Fig. 3:**
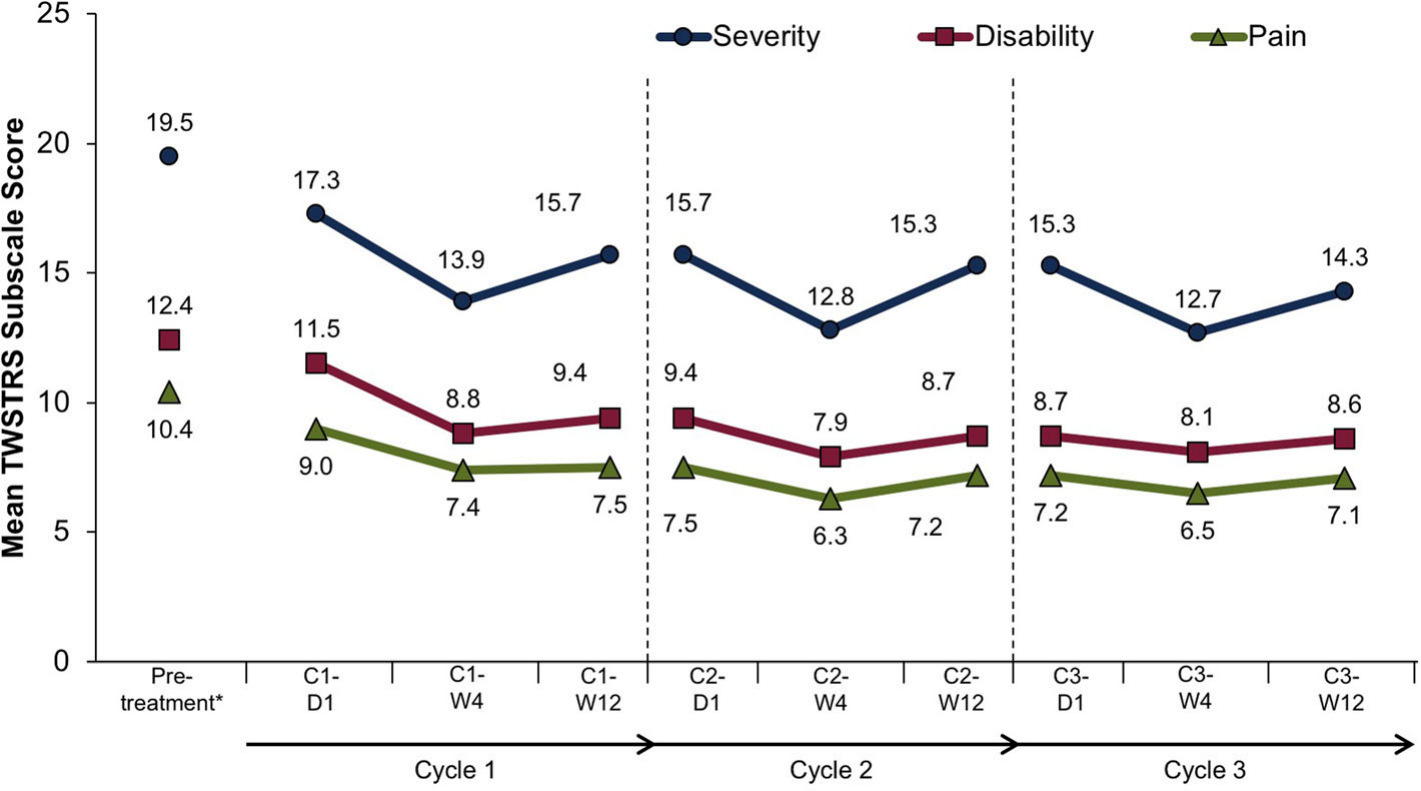
Change from baseline in TWSTRS subscale scores. C, cycle; D, day; W, week. *Before first abobotulinumtoxinA administration

The mean TWSTRS total score was 42.2 (SD 10.3) at baseline in the lead-in study (before first abobotulinumtoxinA administration). In the OLE study, the mean baseline (cycle 1, day 1) TWSTRS total score was 37.7 (SD 13.6) and declined to 30.1 (SD 12.8) at the cycle 3, week 12 visit. Thus, the mean change from the lead-in baseline was − 11.7 (SD 11.2) at cycle 3, week 12 (Fig. 2). For each cycle, TWSTRS total and subscale scores decreased from day 1 to week 4 and increased between week 4 and week 12, though the week 12 scores remained lower than day 1 scores (Figs. 2 and 3). The mean TWSTRS scores at weeks 4 and 12 appeared to decrease for each successive cycle, with no return to baseline (Figs. 2 and 3).

## Discussion

This study demonstrated that abobotulinumtoxinA injected at approved doses using a 2-mL dilution was safe and well tolerated while providing sustained improvements within and across treatment cycles in patients with CD. Most TEAEs reported were mild to moderate in severity and most were considered unrelated to treatment. Dysphagia, a common treatment-related side effect of all available botulinum toxin treatments for CD [9], occurred in 10.7% of patients treated in this study, slightly less than the 15% incidence reported in abobotulinumtoxinA clinical trials [10]. We speculate that this may be attributable to the fact that the majority of patients enrolled in the OLE (77/112) had previously been treated with toxin during the lead-in study, and some may have reached treatment “optimization” during the OLE. A recently published single-site retrospective cohort study [11] in which patients receiving optimized treatment for CD reported lower rates of dysphagia supports this hypothesis. In that study the rates of dysphagia were also unrelated to total dose or dosing of the sternocleidomastoid muscle [11]. In the present study, dysphagia occurring in one patient (<1%) with a history of dysphagia was considered to be a serious treatment-related adverse event, consistent with the incidence of severe dysphagia reported in <5% of cases [9]. There were no discontinuations due to TEAEs. No new safety signals were detected in individuals treated for up to four cycles.

Consistent with the abobotulinumtoxinA pharmacological effect, 4-week efficacy was numerically greater than 12-week efficacy for each cycle. Results were consistent with the lead-in double-blind study and showed that abobotulinumtoxinA using a 2-mL dilution remained effective and well tolerated over four treatment cycles. Moreover, safety and efficacy with abobotulinumtoxinA using the 2-mL dilution were similar to those with 1-mL dilution.

## Conclusions

Extended treatment of cervical dystonia with abobotulinumtoxinA over multiple treatment cycles using a 2-mL dilution is effective and has a positive risk-benefit profile while providing greater dosing flexibility to meet individual patient needs.

## Data Availability

Where patient data can be anonymised, Ipsen will share all individual participant data that underlie the results reported in this article with qualified researchers who provide a valid research question. Study documents, such as the study protocol and clinical study report, are not always available. Proposals should be submitted to DataSharing@Ipsen.com and will be assessed by a scientific review board. Data are available beginning 6 months and ending 5 years after publication; after this time, only raw data may be available.

## Abbreviations

BMI: Body mass index
C: Cycle
CD: Cervical dystonia
D: Day
OLE: Open-label extension
FDA: U.S. Food and Drug Administration
ICH: International Conference on Harmonisation
SAE: Serious adverse event
SD: Standard deviation
TEAE: Treatment-emergent adverse event
TWSTRS: Toronto Western Spasmodic Torticollis Rating Scale
U: Unit
USA: United States of America
W: Week

## References

[1] Dashtipour K, Lew M, Stacy MA (2012). Cervical dystonia. Handbook of Dystonia.

[2] Defazio G, Jankovic J, Giel JL, Papapetropoulos S (2013). Descriptive epidemiology of cervical dystonia. Tremor Other Hyperkinet Mov (N Y).

[3] Marras C, Van den Eeden SK, Fross RD, Benedict-Albers KS, Klingman J, Leimpeter AD, Nelson LM, Risch N, Karter AJ, Bernstein AL, Tanner CM (2007). Minimum incidence of primary cervical dystonia in a multiethnic health care population. Neurology.

[4] Truong D, Duane DD, Jankovic J, Singer C, Seeberger LC, Comella CL, Lew MF, Rodnitzky RL, Danisi FO, Sutton JP (2005). Efficacy and safety of botulinum type a toxin (Dysport) in cervical dystonia: results of the first US randomized, double-blind, placebo-controlled study. Mov Disord.

[5] Truong D, Brodsky M, Lew M, Brashear A, Jankovic J, Molho E, Orlova O, Timerbaeva S (2010). Long-term efficacy and safety of botulinum toxin type a (Dysport) in cervical dystonia. Parkinsonism Relat Disord.

[6] Hauser RA, Truong D, Hubble J, Coleman C, Beffy JL, Chang S, Picaut P (2013). AbobotulinumtoxinA (Dysport) dosing in cervical dystonia: an exploratory analysis of two large open-label extension studies. J Neural Transm (Vienna).

[7] Lew MF, Brashear A, Dashtipour K, Isaacson S, Hauser RA, Maisonobe P, Snyder D, Ondo W (2018). A 500 U/2 mL dilution of abobotulinumtoxinA vs. placebo: randomized study in cervical dystonia. Int J Neurosci.

[8] Consky E, Lang A, Jankovic J (1994). Clinical assessments of patients with cervical dystonia. Therapy with Botulinum Toxin.

[9] Contarino MF, Van Den Dool J, Balash Y, Bhatia K, Giladi N, Koelman JH, Lokkegaard A, Marti MJ, Postma M, Relja M (2017). Clinical practice: evidence-based recommendations for the treatment of cervical dystonia with botulinum toxin. Front Neurol.

[10] Dysport [package insert]. Cambridge: Ipsen Biopharmaceuticals, Inc.; 2020.

[11] Kutschenko A, Klietz M, Paracka L, Kollewe K, Schulte-Sutum A, Janssen T, Schrader C, Wegner F, Dressler D (2020). Dysphagia in cervical dystonia patients receiving optimised botulinum toxin therapy: a single-center retrospective cohort study. J Neural Transm (Vienna).

